# The role of sleep disorders in the risk for CKD and CKD progression

**DOI:** 10.1093/ckj/sfag098

**Published:** 2026-03-23

**Authors:** Carmine Zoccali, Francesca Mallamaci, Mehmet Kanbay, Guido Grassi, Giuseppe Mancia

**Affiliations:** Renal Research Institute, NY, USA; Institute of Molecular Biology and Genetics (Biogem), Ariano Irpino, Italy; Associazione Ipertensione Nefrologia Trapianto Renal (IPNET), c/o Nefrologia, Grande Ospedale Metropolitano, Reggio Calabria, Italy; Associazione Ipertensione Nefrologia Trapianto Renal (IPNET), c/o Nefrologia, Grande Ospedale Metropolitano, Reggio Calabria, Italy; Department of Internal Medicine, Division of Nephrology, Koc University School of Medicine, Istanbul, Turkey; Department of Medicine and Surgery, University of Milan Bicocca, Milan, Italy; University of Milano-Bicocca (Emeritus Professor), Milan, Italy

**Keywords:** chronic kidney disease, insomnia, obstructive sleep apnoea, restless legs syndrome, sleep disorders

## Abstract

Chronic kidney disease (CKD) is a major global health problem and an important driver of cardiovascular morbidity and mortality. Sleep disorders are highly prevalent in people with, or at risk of, CKD, but their specific contribution to CKD onset and progression has not been clearly defined. Unlike previous reviews that have focused mainly on symptom burden, quality of life, or general management of sleep problems in CKD, this narrative review is explicitly centred on renal outcomes. We examine whether common sleep disorders—insomnia, abnormal sleep duration, restless legs syndrome, periodic limb movement disorder, and obstructive sleep apnoea (OSA) and central sleep apnoea—are associated with an increased risk of incident CKD and with faster progression of established CKD [estimated glomerular filtration rate (eGFR) decline, albuminuria, end–stage kidney disease]. We synthesize evidence from prospective cohorts, administrative databases, and Mendelian randomization studies, with particular attention to residual confounding, incomplete sleep phenotyping, and overlap between sleep disorders, especially unrecognized OSA. Observational data suggest that poor sleep quality and abnormal sleep duration are modestly associated with incident CKD and CKD progression, although independence from OSA remains uncertain. In contrast, evidence linking OSA to reduced eGFR, albuminuria, and accelerated CKD progression is more consistent, and bidirectional relations between CKD and OSA are increasingly recognized. We also review pathophysiological pathways that plausibly connect sleep disorders to renal injury and critically appraise preliminary interventional data on OSA treatment and kidney outcomes. Finally, we outline the clinical implications of integrating outcome–oriented sleep assessment into nephrology and hypertension care and propose a research agenda to determine whether systematic detection and treatment of sleep disorders, particularly OSA, should be adopted as a strategy to prevent CKD onset and slow CKD progression.

Chronic kidney disease (CKD), defined as a sustained reduction in glomerular filtration rate (GFR) or evidence of kidney damage for at least 3 months, is a major global health problem, affecting an estimated 800 million people worldwide [1]. CKD has a profound impact on health, both as a direct cause of morbidity and mortality and as a powerful risk factor for cardiovascular disease [2]. Sleep disturbances are highly prevalent in the general population and affect both the quantity and quality of sleep across all age groups [3]. Insomnia, fragmented sleep, obstructive sleep apnoea (OSA), central sleep apnoea (CSA), restless legs syndrome (RLS), periodic limb movement disorder (PLMD), and irregular sleep–wake schedules are increasingly recognized not only as sources of reduced well-being, but also as important contributors to cardiometabolic [4] and renal risk [5]. Emerging evidence suggests that chronic sleep disruption may play a role in the development and progression of CKD [6]. Understanding and addressing sleep disturbances may therefore represent a relevant and potentially modifiable factor in the prevention and management of CKD.

The specific focus of this review is on renal outcomes. We examine whether common sleep disorders are associated with an increased risk of incident CKD and with faster progression of established CKD, beyond the effects of shared cardiometabolic risk factors. In particular, we consider insomnia, RLS, PLMD, and sleep apnoea (OSA and CSA), and their relationships with incident CKD, decline in estimated GFR (eGFR), albuminuria, and end-stage kidney disease (ESKD). Distinct from previous reviews that have primarily emphasized symptom burden and general management of sleep problems in CKD, we focus on kidney outcomes and critically evaluate epidemiological, genetic, and interventional evidence for a causal role of sleep disorders in CKD incidence and progression.

## METHODS AND SCOPE OF THE LITERATURE CONSIDERED

The present work is a narrative (unsystematic) overview of the literature on sleep disorders and their association with incident CKD and CKD progression. We aimed to identify observational and interventional studies, as well as systematic reviews, that reported renal outcomes in relation to sleep disorders in adults. We searched PubMed from database inception to 30 December 2025 using combinations of MeSH terms and free–text keywords related to ‘sleep disorders’, ‘sleep duration’, ‘sleep quality’, ‘insomnia’, ‘obstructive sleep apnea’, ‘central sleep apnea’, ‘restless legs syndrome’, ‘periodic limb movement disorder’, ‘chronic kidney disease’, ‘kidney function decline’, ‘albuminuria’, and ‘end-stage kidney disease’. Reference lists of relevant articles and reviews were also screened to identify additional studies. We considered peer–reviewed original studies, systematic reviews, and key narrative reviews in English that included adult populations (≥18 years) and reported at least one renal outcome relevant to CKD incidence or progression, such as incident CKD, change in eGFR, eGFR slope, change in albuminuria, or incident ESKD. Case reports, small case series (fewer than 20 participants), paediatric studies, and papers reporting only the prevalence of sleep symptoms without renal outcomes were excluded. When multiple reports originated from the same cohort, priority was given to the most significant and/or most recent publication with renal endpoints to avoid double–counting evidence. Titles and abstracts were initially screened by one reviewer (C.Z.), followed by full–text assessment of potentially relevant articles by two authors (C.Z. and F.M.). Study selection was based on conceptual relevance, recency, and overall methodological robustness, with particular attention to the assessment of sleep disorders (e.g. use of polysomnography versus questionnaires), definition of renal outcomes, and adjustment for key confounders. Data were extracted in a non–standardized manner and synthesized narratively, grouping findings into major themes: (i) insomnia, RLS, and PLMD; (ii) OSA and CSA; (iii) intermediate pathophysiological mechanisms; and (iv) interventional studies targeting sleep disorders with renal endpoints.

Given the use of a single bibliographic database, single–reviewer screening at the title/abstract level, and the absence of a formal risk–of–bias assessment or quantitative meta–analysis, this review should be regarded as narrative and hypothesis–generating rather than a definitive, comprehensive synthesis.

## SLEEPING DISTURBANCES IN CKD

Insomnia, a disorder characterized by difficulty initiating or maintaining sleep or by early morning awakening with daytime impairment, is observed in both early and advanced CKD and is consistently associated with a reduction in quality of life [5]. In CKD, insomnia is often multifactorial: metabolic and endocrine abnormalities, uremic symptoms such as pruritus, muscle cramps, neuropathic pain, and bone pain, systemic inflammation, and psychological distress all contribute to sleep fragmentation. Medications commonly used in CKD, including β-agonists, β-blockers, antidepressants, diuretics, and immunosuppressive drugs, may further disturb sleep through central effects, circadian shifts, or nocturnal diuresis [5].

OSA [5] and, less frequently [7], CSA [8] are common sleep-related breathing disorders in CKD. Repeated episodes of upper airway obstruction characterize OSA during sleep, whereas CSA involves transient interruptions of the central respiratory drive. These events often coexist and lead to intermittent hypoxia, marked intrathoracic pressure swings, frequent arousals, and sleep fragmentation. In CKD, chronic volume overload, nocturnal rostral fluid shift, pharyngeal oedema, pulmonary congestion, and uremic neuropathy or myopathy all favour upper airway collapsibility and ventilatory instability, predisposing to both obstructive and central apnoeas.

RLS is defined by an uncontrollable urge to move the legs, usually accompanied by unpleasant sensations that worsen at rest and in the evening and are relieved by movement. RLS can severely disrupt sleep, causing insomnia and daytime sleepiness, and is associated with increased depressive symptoms and reduced quality of life in CKD [9]. PLMD, characterized by involuntary, repetitive limb movements during sleep, often occurs in conjunction with RLS and further fragments sleep architecture, leading to nonrestorative sleep and excessive daytime sleepiness. Both RLS and PLMD are more prevalent in advanced CKD and in dialysis patients, implicating uremic toxins, iron deficiency, altered dopaminergic neurotransmission, and chronic inflammation in their pathogenesis [10].

## INSOMNIA, RLS, AND PLMD IN RELATION TO CKD INCIDENCE AND PROGRESSION

Several large cohort studies have investigated whether insomnia symptoms, abnormal sleep duration, and related disturbances predict incident CKD [11]. In 2019, Bo *et al*. analysed a cohort of 194 039 Chinese adults without CKD aged ≥20 years, in whom they constructed a composite sleep score combining sleep duration and sleep quality. Participants were followed for incident CKD. Compared with those with the most favourable sleep scores (>6), individuals with intermediate (4–6) and poor (<4) scores had progressively higher risks of incident CKD, with hazard ratios of approximately 1.07 and 1.61, respectively. The analysis adjusted for a broad range of cardiometabolic and lifestyle covariates. However, OSA was not formally diagnosed or excluded by polysomnography; thus, independence from sleep–disordered breathing relies on adjustment for proxies such as body mass index (BMI), snoring symptoms and comorbidities, and residual confounding by unrecognized OSA cannot be ruled out.

Earlier, Choi *et al*. [12] used data from the Korean Genome and Epidemiology Study (KoGES–Kangwha study) to examine the association between self–reported sleep duration and incident CKD in Korean adults. In this cohort, longer—rather than shorter—sleep duration was associated with increased CKD incidence in women but not in men. Again, sleep was assessed by questionnaire, and there was no systematic polysomnographic screening for OSA, so confounding by unrecognized sleep–disordered breathing is possible.

A prospective cohort study of Japanese workers [13] followed 3600 enrollees for a mean of 4.4 years to assess the relationship between insomnia symptoms, shift work, and incident CKD, defined by a decline in eGFR. In this study, awakening during the night—essentially a marker of insomnia—was associated with a moderately increased risk of incident CKD after adjustment for age, baseline eGFR, hypertension, and other clinical variables. However, exposure assessment again relied on questionnaires, and there was no systematic screening or polysomnography to exclude OSA, leaving room for residual confounding by unrecognized sleep–disordered breathing.

Turning from incident CKD to CKD progression [14], Yamamoto *et al*. studied 1601 patients with established CKD. They found that self–reported sleep duration and sleep quality were associated with progression to ESKD. They reported a U–shaped relationship between sleep duration and risk of progression, with individuals sleeping <5 h or >8 h per night at greatest risk. Poor sleep quality, assessed using the Pittsburgh sleep quality index (PSQI), was also associated with higher ESKD incidence. As in the incident CKD studies, sleep was self–reported and OSA was not systematically ruled out; multivariable models likely adjusted for obesity and cardiovascular disease, but complete independence of these associations from OSA cannot be demonstrated.

Mendelian randomization offers a complementary approach that, in principle, is less vulnerable to confounding by lifestyle and comorbidities. One Mendelian randomization study [15] examined genetic proxies for sleep traits in approximately 337 000 unrelated individuals from the UK Biobank in relation to ESKD. This analysis yielded suggestive associations for some sleep traits, but the overall causal signal was modest and did not support a strong, specific effect of genetically instrumented insomnia on ESKD risk. Accordingly, while the findings are compatible with a modest causal contribution of adverse sleep traits, they are insufficient to establish that insomnia *per se* robustly causes ESKD, independent of intermediate mechanisms or coexisting sleep disorders.

A 2022 systematic review and meta–analysis in patients with CKD collated observational data on sleep quality and insomnia [16]. Approximately half of patients with advanced CKD had poor sleep quality or insomnia, with even higher prevalence among those receiving kidney replacement therapy. However, most included studies were cross–sectional, very few had robust longitudinal designs with renal endpoints, and almost none could isolate the effect of insomnia independent of coexisting OSA. The authors therefore concluded that insomnia should be regarded primarily as a highly prevalent comorbidity and a major contributor to symptom burden and impaired quality of life, rather than as a proven causal driver of eGFR decline. This interpretation is echoed by recent narrative reviews [10, 17], which emphasize cardiovascular, metabolic, and psychosocial sequelae more than kidney–specific causality.

For RLS, the evidence is even further from establishing a direct causal effect on incident CKD or CKD progression independent of sleep apnoea. An updated systematic review by Safarpour *et al*. [18] noted that RLS is two– to three–fold more common in CKD than in the general population and that CKD patients with RLS have higher mortality and higher rates of cardiovascular events, depression, insomnia, and impaired quality of life than CKD patients without RLS. This pattern supports RLS as a marker and mediator of symptom burden and cardiovascular risk in CKD, but the review did not present convincing longitudinal data showing that RLS in otherwise healthy individuals leads to incident CKD, or that RLS in CKD patients independently accelerates GFR decline after controlling for comorbidities and sleep–disordered breathing.

A meta–analysis focusing on the prevalence of RLS in CKD [19] confirmed high RLS prevalence across CKD stages and dialysis modalities and described an association between CKD and RLS largely in terms of comorbidity patterns rather than as an upstream cause of kidney dysfunction. Individual observational studies, such as a Japanese questionnaire survey of RLS in CKD [20], showed that RLS symptoms correlate with mood disturbance and insomnia and become more frequent with advancing CKD stage. However, this and similar studies lacked serial eGFR measurements and did not report renal outcomes, precluding assessment of whether RLS accelerates CKD progression. Mechanistically, RLS in CKD is closely tied to iron deficiency, uraemia, and dopaminergic alterations, which are themselves consequences of advanced renal failure, further complicating causal inference.

Taken together, these data provide modest prospective evidence that self–reported insomnia symptoms and abnormal sleep duration are associated with increased risk of incident CKD and with faster progression to ESKD. However, in none of these observational studies is OSA rigorously excluded by polysomnography; adjustment for BMI, snoring, and other covariates reduces but does not eliminate the possibility that undiagnosed sleep–disordered breathing partly mediates the observed associations. Mendelian randomization findings are compatible with a small causal component of adverse sleep traits on renal outcomes but fall short of establishing a strong, independent effect [15]. For RLS and PLMD, available evidence mainly supports their role as highly prevalent, symptom–driving comorbidities in CKD, without robust proof that they independently cause incident CKD or accelerate CKD progression.

## CAUSAL ROLE OF OSA IN THE RISK OF CKD

A causal role of CSA in incident CKD or in faster CKD progression remains biologically plausible but unproven, representing a clear research gap [8]. Most available data and the following discussion, therefore, focus on OSA. Relevant studies are summarized in Table 1 and briefly outlined below.

**Table 1a:** Cross-sectional studies evaluating sleep apnoea/sleep quality and renal function.

Ref.	Population/setting	Design/sleep assessment	Renal outcome	Key findings
[21]	254 CKD pts (eGFR < 60 ml/min/1.73 m^2^, incl. ESKD) from nephrology clinics vs sleep-clinic referrals with eGFR ≥ 60 (Alberta Kidney Network)	Cross-sectional comparison; OSA diagnosis and nocturnal hypoxia by standard sleep testing	CKD status; nocturnal hypoxia	In referral pts with preserved kidney function, OSA prevalence was 27% and nocturnal hypoxia 16%, whereas both were substantially more common in CKD pts, underscoring a strong link between impaired kidney function, fluid overload, and sleep-disordered breathing.
[22]	998 adults referred for overnight polysomnography for suspected sleep-disordered breathing	Cross-sectional; polysomnography; OSA severity; blood pressure phenotypes (advanced and resistant HTN)	Presence of CKD	Older age, severe sleep apnoea, stage III hypertension, and resistant hypertension were each independently associated with higher odds of CKD (ORs 3.96, 2.28, 3.55, and 9.42, respectively), suggesting that severe OSA and resistant HTN are independent renal risk factors and that their coexistence identifies a subgroup at particularly high renal risk.
[23]	823 Han Chinese women, 40–67 years, menopause clinic (no CKD enrichment)	Cross-sectional; PSQI (sleep quality), sleep duration, efficiency, latency, disturbances	Renal function by serum Cys-C; decline defined as Cys-C ≥ 0.91 mg/dl	Poor perceived sleep quality, sleep duration <6 h, sleep efficiency <75%, prolonged sleep latency, and more severe sleep disturbances were each associated with >2-fold higher odds of declining renal function after adjustment. Overall sleep disorder (PSQI ≥ 8), late postmenopause, and high Cys-C quartile increased risk, whereas menopausal hormone therapy was associated with lower risk; OSA was not specifically isolated from other sleep traits.

CKD, chronic kidney disease; eGFR, estimated glomerular filtration rate; ESKD, end–stage kidney disease; OSA, obstructive sleep apnoea; HTN, hypertension; PSQI, Pittsburgh sleep quality index; Cys–C, cystatin C; OR, odds ratio.

Cross-sectional studies (Table 1a) initially drew attention to the high prevalence of OSA in CKD and the strong association between OSA severity and markers of renal dysfunction. Nichol *et al*. [21] compared 254 CKD patients (eGFR < 60 ml/min/1.73 m^2^), including those with ESKD, from nephrology clinics in the Alberta Kidney Network with a group of patients referred to a sleep centre for suspected OSA who had normal or near-normal renal function (eGFR ≥ 60 ml/min/1.73 m^2^). In the referral population with preserved kidney function, OSA prevalence was 27% and nocturnal hypoxia occurred in 16%, whereas in the CKD population, both OSA and nocturnal hypoxia were substantially more common, underscoring the close link between impaired kidney function, fluid overload, and sleep-disordered breathing.

The interplay between sleep apnoea and blood pressure complicates interpretation of their combined effects on CKD. In a cross-sectional cohort of 998 subjects undergoing overnight polysomnography for suspected sleep-disordered breathing [22], older age, severe sleep apnoea, advanced hypertension, and resistant hypertension were each independently associated with higher odds of CKD. In fully adjusted models, participants aged ≥65 years had an odds ratio for CKD of 3.96 (95% CI 2.57–6.09; *P* < 0.001). Severe sleep apnoea conferred an odds ratio of 2.28 (95% CI 1.13–4.58; *P* < 0.05), stage III hypertension an odds ratio of 3.55 (95% CI 1.70–7.42; *P* < 0.001), and resistant hypertension an odds ratio of 9.42 (95% CI 4.22–21.02; *P* < 0.001). These findings suggest that severe sleep apnoea and resistant hypertension may act as independent renal risk factors, and that their coexistence identifies a subgroup at particularly high risk of kidney damage.

Associations between sleep characteristics and renal function have also been observed in non-CKD populations. In a cross-sectional analysis of 823 Han Chinese women aged 40–67 years attending a menopause clinic [23], sleep quality was measured by the PSQI and renal function by serum cystatin C (Cys-C). After adjustment for potential confounders, poor perceived sleep quality, sleep duration <6 h, sleep efficiency <75%, prolonged sleep latency, and more severe sleep disturbances were each associated with more than a two-fold increase in the odds of declining renal function, defined as Cys-C ≥ 0.91 mg/dl, in postmenopausal women. Overall sleep disorder (PSQI ≥ 8), late postmenopausal status, and being in the highest quartile of Cys-C independently increased the odds of declining renal function, whereas menopausal hormone therapy was associated with a substantially lower likelihood of decline. These findings support a link between global sleep disruption and reduced renal function, although they do not distinguish the specific contribution of OSA from that of other sleep traits.

Longitudinal cohort studies (Table 1b) have provided more direct evidence that OSA is associated with CKD risk. In 2012, Kanbay *et al*. [24] retrospectively evaluated 175 subjects admitted to a sleep disorders centre. Participants were classified by apnoea–hypopnoea index (AHI) as OSA-negative (AHI < 5), mild (5–15), moderate (15–30), or severe (>30). The prevalence of diabetes, hypertension, and higher BMI was greatest in those with severe OSA. A significant, graded decline in GFR was observed across OSA severity categories, from 50 ± 12 ml/min in those with normal AHI to 39 ± 16 ml/min in those with AHI > 30. This trend remained statistically significant after adjustment for hypertension, diabetes, age, and sex (*P* = 0.02), suggesting that more severe OSA is associated with worse kidney function, even after accounting for traditional risk factors.

**Table 1b:** Longitudinal, cohort, and Mendelian randomization studies on sleep apnoea/sleep traits and kidney outcomes.

Ref.	Population/setting	Design/sleep assessment	Renal outcome/follow-up	Key findings
[24]	175 adults admitted to a sleep disorders centre	Retrospective cohort; AHI categories: <5, 5–15, 15–30, >30 events per h	GFR across OSA severity	Prevalence of diabetes, hypertension, and higher BMI was greatest in severe OSA. There was a graded decline in GFR from 50 ± 12 ml/min (AHI < 5) to 39 ± 16 ml/min (AHI > 30); this trend remained significant after adjustment for age, sex, hypertension, and diabetes (*P* = 0.02), supporting an association between increasing OSA severity and lower kidney function beyond traditional risk factors.
[25]	1525 adults in the ARIC cohort, free of CKD at baseline	Prospective community-based cohort; polysomnography; OSA severity	Incident CKD stage ≥3 over median 19 years	Severe OSA was associated with higher risk of incident CKD, but the association was attenuated and lost statistical independence after adjustment for BMI and related factors, highlighting substantial confounding by obesity and other shared cardiometabolic risks.
[26]	1295 adults referred to five Canadian academic sleep centres for suspected OSA	Prospective observational cohort; AHI-based categories (no/mild, moderate, severe OSA)	KDIGO risk categories for CKD progression (by eGFR and ACR)	Moderate-to-very high progression risk was present in 13.6%, 28.9%, and 30.9% of the no/mild, moderate, and severe OSA groups (*P* < 0.001). After multivariable adjustment, ORs for moderate-to-very high risk were 2.63 (moderate) and 2.96 (severe) vs no/mild OSA. Among those at increased risk, 73% were unaware of abnormal kidney function, underscoring the potential of sleep clinics as sites for CKD detection.
[27]	1657 MESA sleep participants, including 287 with CKD at baseline	Longitudinal cohort (∼5-year follow-up); polysomnography, actigraphy, and questionnaires; high-dimensional regression	ΔeGFR and ΔUACR over follow-up	In baseline CKD, faster eGFR decline was associated with reduced N3 sleep, more daytime napping, and a later sleep midpoint trajectory. Increases in UACR were linked to more nocturnal wake bouts and greater sleep-related hypoxaemia (≥5% of sleep time with SpO₂ < 90%). Several sleep parameters (N3, napping, sleep midpoint) modified the association between HbA1c and eGFR decline, suggesting sleep traits modulate the impact of glycaemic burden on kidney function.
[28]	754 consecutive pts without CKD at enrolment in the HSCAA study; 231 with diabetes	Prospective cohort; sleep apnoea, subjective sleep quality, and heart rate variability	Renal outcome defined by eGFR decline	In pts with diabetes, poor sleep quality and low heart rate variability—but not OSA *per se—w*ere associated with higher renal risk. Poor sleep quality remained independently associated with eGFR decline (HR 2.57; 95% CI 1.01–6.53; *P* = 0.045) after adjustment for sleep apnoea and conventional risk factors. In nondiabetics, poor sleep quality similarly conferred increased renal risk, whereas sleep apnoea and low heart rate variability were not significant predictors.
[29]	Taiwan NHIRD: adults ≥30 years with newly diagnosed sleep apnoea (2000–2010); nationwide coverage >96%	Retrospective administrative cohort; sleep apnoea vs matched controls	Incident CKD and ESKD through 2011	Sleep apnoea was associated with nearly double the incidence of CKD (HR 1.94; 95% CI 1.52–2.46; *P* < 0.001) and a 2.2-fold increase in ESKD incidence (95% CI 1.31–3.69; *P* < 0.01) vs controls, independent of age, sex, and comorbidities. These findings, in a country with very high ESKD incidence and prevalence, support a clinically relevant link between sleep apnoea and adverse kidney outcomes in real-world practice.
[30]	Multicentre Canadian Sleep and Circadian Network cohort	Observational cohort; AHI, event-related hypoxic burden, and other physiologic metrics	Cardiovascular and neurophysiologic responses; implications for renal/CV outcomes	Higher event-related hypoxic burden was associated with greater postevent increases in heart rate, vasoconstriction, and cortical β-power, indicating amplified cardiovascular and neurophysiologic responses to respiratory events. These data suggest that composite measures incorporating depth and duration of desaturation, together with response variability, may provide more biologically relevant indices of OSA severity than AHI alone and could better correlate with renal and cardiovascular outcomes.
[31]	1589 259 Japanese adults with routine health check-ups; no prior sleep apnoea diagnosis or renal replacement therapy	Large retrospective cohort; baseline eGFR and proteinuria as exposures; incident sleep apnoea as outcome	Incident sleep apnoea over median ∼3.2 years	During follow-up, 11 054 participants (0.7%) developed sleep apnoea. Progressively lower eGFR was associated with higher risk of incident sleep apnoea in a dose–response pattern, with adjusted HRs 1.13, 1.22, 1.34, and 1.82 for eGFR 60–89, 45–59, 30–44, and <30 vs ≥90 ml/min/1.73 m^2^, respectively. Proteinuria modestly increased sleep apnoea risk. Findings support reduced kidney function as an independent risk factor for incident sleep apnoea.
[32]	FinnGen Consortium: 375 657 participants (38 998 OSA cases; 336 659 controls)	Two-sample Mendelian randomization; genetic instruments for OSA and seven renal phenotypes; bidirectional analysis	Genetic causal effects of OSA on renal traits and of renal traits on OSA	No significant causal effect of genetically predicted OSA on renal function traits was detected. Conversely, after outlier removal, genetically predicted increases in blood urea nitrogen were associated with higher OSA risk, supporting a causal contribution of impaired renal function to OSA but not a strong direct causal effect of OSA on kidney function.

ACR, albumin-to-creatinine ratio; AHI, apnoea–hypopnoea index; ARIC, Atherosclerosis Risk in Communities; BMI, body mass index; CKD, chronic kidney disease; CV, cardiovascular; eGFR, estimated glomerular filtration rate; ESKD, end-stage kidney disease; HbA1c, glycated haemoglobin; HR, hazard ratio; HSCAA, Hyogo Sleep Cardio-Autonomic Atherosclerosis; KDIGO, Kidney Disease: Improving Global Outcomes; MESA, Multi-Ethnic Study of Atherosclerosis; NHIRD, National Health Insurance Research Database; OSA, obstructive sleep apnoea; pts, patients; SpO_2_, oxygen saturation; UACR, urinary albumin-to-creatinine ratio.

In the ARIC (Atherosclerosis Risk in Communities) cohort [25], polysomnography data from 1525 adults free of CKD at baseline were used to examine the association between OSA and incident CKD (stage ≥3) over a median follow-up of 19 years. Severe OSA was associated with increased risk of incident CKD, but this association was not independent of obesity; after adjustment for BMI and related factors, the excess risk was attenuated, highlighting the challenge of separating the effects of OSA from those of shared risk factors such as obesity.

A large prospective observational study by Beaudin *et al*. [26] examined CKD risk in 1295 adults referred to five Canadian academic sleep centres for suspected OSA. AHI categorized patients as no/mild, moderate, or severe OSA. A moderate-to-very high risk of CKD progression—defined by reduced eGFR, elevated albumin-to-creatinine ratio, or both—was present in 13.6%, 28.9%, and 30.9% of these groups, respectively (*P* < 0.001). After adjustment for established CKD risk factors, the odds ratios for moderate-to-very high CKD progression risk were 2.63 (95% CI 1.79–3.85) for moderate OSA and 2.96 (95% CI 2.04–4.30) for severe OSA compared with no/mild OSA. Notably, among patients at increased risk of CKD progression, 73% were unaware of having abnormal kidney function, underscoring both the silent nature of early CKD and the potential utility of sleep clinics as points of CKD detection.

Chen *et al*. [27] investigated associations between detailed sleep characteristics and longitudinal changes in kidney function in 1657 participants from the MESA Sleep cohort, 287 of whom had CKD at baseline. Sleep measures derived from polysomnography, actigraphy, and questionnaires, along with cardiovascular risk factors, were related to changes in eGFR and urinary albumin-to-creatinine ratio (UACR) over approximately five years using high-dimensional regression methods. Among participants with CKD at baseline, a faster decline in eGFR was associated with reduced N3 (deep) sleep, more frequent daytime naps, and a later sleep midpoint trajectory. In contrast, increases in UACR were associated with a high number of nocturnal wake bouts and greater sleep-related hypoxaemia (at least 5% of sleep time with oxygen saturation <90%). Several sleep parameters, including N3 sleep, napping behaviour, and sleep midpoint, significantly modified the association between HbA1c and eGFR decline, suggesting that sleep traits may modulate the impact of glycaemic burden on kidney function.

In the HSCAA (Hyogo Sleep Cardio-Autonomic Atherosclerosis) study [28], 754 consecutive patients without CKD, 231 of whom had diabetes, were enrolled to examine relationships between sleep apnoea, sleep quality, autonomic imbalance, and renal function decline. Among patients with diabetes, poor sleep quality and low heart rate variability—but not sleep apnoea *per se*—were associated with a significantly higher risk of reaching a renal outcome. In multivariable models, poor sleep quality remained significantly associated with increased risk of eGFR decline (hazard ratio 2.57; 95% CI 1.01–6.53; *P* = 0.045), independent of sleep apnoea and conventional risk factors. In nondiabetic patients, poor sleep quality likewise conferred increased renal risk, whereas sleep apnoea and low heart rate variability were not significant predictors.

A retrospective cohort study using NHIRD (Taiwan’s National Health Insurance Research Database) [29], which covers > 96% of the Taiwanese population, followed adults (age ≥30 years) with newly diagnosed sleep apnoea between 2000 and 2010 through 2011. Patients with sleep apnoea had nearly double the incidence of CKD (hazard ratio 1.94; 95% CI 1.52–2.46; *P* < 0.001) and a 2.2-fold increase in ESKD incidence (95% CI 1.31–3.69; *P* < 0.01) compared with matched controls, independent of sex, age, and comorbid conditions. These findings are notable given Taiwan’s very high incidence and prevalence of ESKD and lend support to a link between sleep apnoea and adverse kidney outcomes in real-world practice.

More refined measures of OSA severity may be more informative than the AHI alone. In a multicentre Canadian Sleep and Circadian Network cohort [30], patients with higher event-related hypoxic burden exhibited greater post-event increases in heart rate, vasoconstriction, and cortical β-power, indicating amplified cardiovascular and neurophysiological responses. These observations suggest that integrating the depth and duration of desaturation events, together with the variability of physiological responses, may yield more biologically relevant metrics of OSA severity that better correlate with renal and cardiovascular outcomes.

The bidirectional nature of the OSA–CKD relationship is supported by a large Japanese retrospective cohort of 1589 259 adults [31] with health check-up data and no prior diagnosis of sleep apnoea or renal replacement therapy. Over a median follow-up of about 3.2 years, 11 054 participants (0.7%) developed sleep apnoea. After adjustment for demographic and cardiometabolic factors, progressively lower eGFR was associated with higher risk of incident sleep apnoea in a clear dose–response pattern, with adjusted hazard ratios of 1.13, 1.22, 1.34, and 1.82 for eGFR 60–89, 45–59, 30–44 and <30 ml/min/1.73 m^2^, respectively, compared with ≥90 ml/min/1.73 m^2^. Proteinuria modestly increased sleep apnoea risk. These findings support reduced kidney function as an independent risk factor for incident sleep apnoea, reinforcing CKD–OSA association.

Finally, a two-sample Mendelian randomization study using genetic data from the FinnGen Consortium [32] (375 657 participants; 38 998 OSA cases and 336 659 controls) investigated causal relations between OSA and seven renal function phenotypes. This analysis did not detect a significant causal effect of genetically predicted OSA on renal function traits. Conversely, after outlier removal, genetically predicted increases in blood urea nitrogen were associated with higher OSA risk. Thus, in this European population, Mendelian randomization did not support a causal effect of OSA on renal function but did support an effect of impaired renal function on OSA risk.

Overall, the convergence of evidence from cross-sectional studies, large population-based cohorts, and genetic analyses supports a close association between OSA and CKD. Observational data consistently show that OSA is associated with increased CKD incidence, faster eGFR decline, albuminuria, and excess cardiovascular risk, particularly in high-risk populations such as those with diabetes, hypertension, or obesity. However, shared risk factors—most notably obesity and fluid overload—and residual confounding continue to complicate causal inference, and Mendelian randomization to date does not confirm a strong, direct causal effect of OSA on kidney function.

## DOES OSA THERAPY IMPACT CKD PROGRESSION? INTERVENTIONAL EVIDENCE

Interventional evidence on whether treating OSA improves renal outcomes remains limited. Most available data are retrospective or observational and involve heterogeneous populations, small sample sizes, and relatively short follow–up, which restrict causal inference (Table 2).

**Table 2:** Interventional and treatment studies of OSA and renal outcomes.

Ref.	Population/setting	Design and OSA treatment	Renal outcome/follow-up	Main findings and key limitations
[33]	32 220 CKD pts with incident OSA (Taiwan LGTD2005, 2000–2016)	Large retrospective cohort using claims data; among CKD pts with incident OSA, 1078 underwent OSA surgery vs 3234 propensity score-matched OSA pts without surgery	Incident ESKD and mortality; competing-risk analyses accounting for death	Surgical group had significantly lower ESKD incidence (adjusted HR 0.38; 95% CI 0.15–0.97; *P* = 0.043), suggesting reduced long-term progression to ESKD. However, overall and non-CV mortality were markedly higher in the surgical cohort (HR 2.54 and 2.62, both *P* < 0.0001), largely due to excess deaths in the first three postoperative months. Results imply potential renoprotection but at the cost of substantial short-term mortality; residual confounding and lack of granular clinical data limit causal inference and patient-selection guidance.
[34]	38 men with OSA (polysomnography-diagnosed); baseline eGFR 77.3 ± 12.0 ml/min/1.73 m^2^	Small prospective, uncontrolled interventional study; 3 months of nasal CPAP	Change in eGFR over 3 months	In multivariable analyses, older age and longer mean apnoea duration independently predicted lower eGFR. After 3 months of CPAP, eGFR increased significantly, suggesting a potentially reversible component of OSA-related kidney dysfunction in the short term. Interpretation is limited by small sample size, absence of a control group, short follow-up, and possible regression to the mean and Hawthorne effects.
[35]	395 pts with stage G3–G4 CKD and OSA	Nonrandomized cohort; 52 OSA pts elected 12-month nasal CPAP, remaining pts received usual care without CPAP	Rate of eGFR decline over 12 months	CPAP use was associated with slower eGFR decline compared with non-CPAP, particularly among pts with moderate-to-severe OSA, suggesting a potential renoprotective effect. However, substantial selection bias (self-selection into CPAP), small treated group, residual confounding, and nonrandomized design markedly weaken causal conclusions.
[36]	42 pts with stage 3–5 CKD and OSA	Retrospective cohort; exposure defined by CPAP adherence (>4 h/night on >70% nights vs poorer adherence)	eGFR trajectory and proteinuria	Good CPAP adherence was associated with minimal eGFR decline and lower proteinuria compared with poor adherence, consistent with a dose–response-type relationship between effective OSA treatment and kidney outcomes. Nonetheless, very small sample size, wide confidence intervals, retrospective design, and potential ‘healthy-adherer’ bias limit precision and generalizability.
[37, 38]	Adults with CKD and OSA from nephrology clinics (target sample: 1665 eligible; only 57 randomized)	12-month randomized, controlled, nonblinded CPAP trial vs usual care	Intended outcome: effect of CPAP on kidney function decline over 12 months	Over 3 years, only 57/1665 eligible CKD pts (3.4%) were randomized due to nonattendance, recruitment constraints, prior OSA treatment, refusal of consent, and incomplete sleep testing. The trial was underpowered and feasibility-limited, preventing robust conclusions about CPAP’s renal effects. The study mainly demonstrates major practical barriers to adequately powered RCTs of CPAP in largely asymptomatic CKD patients with OSA.
[39]	469 elderly outpatients (mean age 74.4 ± 5.3 years) with newly diagnosed moderate-to-severe OSA and excessive daytime sleepiness; single centre	Prospective observational study; all pts completed 1-week auto-CPAP trial, then managed with ongoing CPAP (adherence >4 h/night; *n* = 210) vs best medical therapy alone (*n* = 259)	RKFD, defined as eGFR loss ≥5 ml/min/1.73 m^2^ per year; mean follow-up 20.7 ± 5.5 months	129 RKFD events occurred; incidence was 8.33 vs 18.20 events per 100 patient-years in CPAP vs untreated groups (log-rank *P* < 0.001). In multivariable Cox models, CPAP independently reduced RKFD risk (HR 0.376; 95% CI 0.254–0.557), suggesting a renoprotective effect in this high-risk elderly cohort. Limitations include nonrandomized, single-centre design; self-selection and adherence biases; relatively short follow-up; incomplete reporting of baseline CKD status and long-term adherence; and reliance on short-term RKFD rather than harder kidney endpoints, limiting mechanistic insight and causal inference.

AHI, apnoea–hypopnoea index; CKD, chronic kidney disease; CV, cardiovascular; eGFR, estimated glomerular filtration rate; ESKD, end-stage kidney disease; HR, hazard ratio; LGTD2005, Longitudinal Generation Tracking Database 2005; OSA, obstructive sleep apnoea; pts, patients; RCT, randomized controlled trial; RKFD, rapid kidney function decline.

A large retrospective cohort study using Taiwan’s 2005 Longitudinal Generation Tracking Database (LGTD2005) [33] analysed claims data from 32 220 CKD patients with incident OSA between 2000 and 2016. Among these, 1078 patients who underwent surgical treatment for OSA were compared with 3234 propensity score-matched patients with incident OSA who did not receive surgery. In competing–risk analyses that accounted for mortality, the incidence of ESKD was significantly lower in the surgically treated group than in the untreated cohort, with an adjusted hazard ratio of 0.38 (95% CI 0.15–0.97; *P* = 0.043), suggesting a markedly reduced long–term risk of progression to ESKD among surgically treated patients. However, overall and non–cardiovascular mortality were substantially higher in the surgical group, with adjusted hazard ratios of 2.54 (95% CI 1.79–3.59; *P* < 0.0001) and 2.62 (95% CI 1.83–3.75; *P* < 0.0001), respectively, due largely to excess deaths in the early postoperative period (first 3 months). These findings indicate that in CKD patients with incident OSA, OSA surgery may lower long–term renal risk but carries substantial short–term mortality risk, calling for careful patient selection and perioperative management.

Several smaller studies have assessed the renal impact of continuous positive airway pressure (CPAP). Koga *et al*. [34] studied 38 men with OSA diagnosed by polysomnography and baseline eGFR 77.3 ± 12.0 ml/min/1.73 m^2^. In multivariable analyses, older age and longer mean apnoea duration independently predicted lower eGFR. After 3 months of nasal CPAP, eGFR increased significantly, suggesting that part of the kidney dysfunction associated with OSA may be reversible with effective therapy in the short term. However, the small sample size, short duration, and lack of a control group limit interpretation.

A nonrandomized cohort study by Li *et al*. [35] evaluated whether 12–month nasal CPAP slows CKD progression in patients with stage G3–G4 CKD and OSA. Among 395 patients, 52 with OSA elected to use CPAP. Compared with non–CPAP patients, those treated with CPAP had slower eGFR decline, particularly in those with moderate–to–severe OSA. Nonetheless, substantial selection bias (patients self–selecting CPAP), small numbers in the treated group, and residual confounding weaken causal conclusions.

In another retrospective cohort study, Puckrin *et al*. [36] examined 42 patients with stage 3–5 CKD and OSA and assessed the association between CPAP adherence and renal outcomes. Good CPAP adherence—defined as use >4 h per night on >70% of nights—was associated with minimal eGFR decline and lower proteinuria compared with poor adherence. However, wide confidence intervals, the small sample size, and potential healthy–adherer bias limit the precision and generalizability of these findings.

Attempts to test CPAP in CKD using randomized designs have faced substantial feasibility challenges. A 12–month, randomized, controlled, nonblinded trial by Rimke *et al*. [37, 38] aimed to evaluate whether CPAP slows kidney function decline in adults with CKD and OSA. Over 3 years, only 57 of 1665 eligible CKD patients (3.4%) were ultimately randomized, owing to multiple barriers including nonattendance, limited recruitment capacity, prior OSA treatment, refusal of consent, and incomplete sleep testing. This experience highlights the practical difficulties of conducting adequately powered randomized controlled trials (RCTs) of CPAP in largely asymptomatic CKD patients with OSA.

A more recent prospective single–centre observational study enrolled 469 elderly outpatients (mean age 74.4 ± 5.3 years) with newly diagnosed moderate–to–severe OSA and excessive daytime sleepiness [39]. After baseline clinical and laboratory assessment, all participants completed a 1–week auto–CPAP trial and were then managed either with ongoing CPAP (adherence >4 h/night; *n* = 210) or best medical therapy alone (*n* = 259). Over a mean follow–up of 20.7 ± 5.5 months, 129 rapid kidney function decline (RKFD) events occurred, defined as eGFR loss ≥5 ml/min/1.73 m^2^ per year: incidence rates were 8.33 versus 18.20 events per 100 patient–years in the CPAP and untreated groups, respectively (log–rank *P* < 0.001). In multivariable Cox models, CPAP independently reduced RKFD risk (hazard ratio 0.376; 95% CI 0.254–0.557), suggesting a potentially renoprotective effect in this high–risk population. However, the nonrandomized, single–centre design, self–selection into CPAP (raising the possibility of healthy–adherer and residual confounding biases), the relatively short follow–up, incomplete description of baseline CKD status and long–term adherence, and the use of RKFD over a short time window as the primary outcome all limit causal inference and mechanistic insight.

Collectively, these observational and preliminary interventional studies support the biological plausibility that OSA contributes to CKD progression and that OSA therapy may favourably affect renal function and proteinuria in selected patients. Nevertheless, definitive evidence from large, methodologically robust RCTs with kidney endpoints is still lacking. Future trials will need to overcome substantial recruitment and adherence challenges and should be designed with rigorous sleep phenotyping, standardized renal outcomes, adjudicated cardiovascular events, and careful monitoring of treatment adherence and tolerability.

## PATHOGENESIS OF SLEEP DISORDERS IN CKD

**Figure 1:**
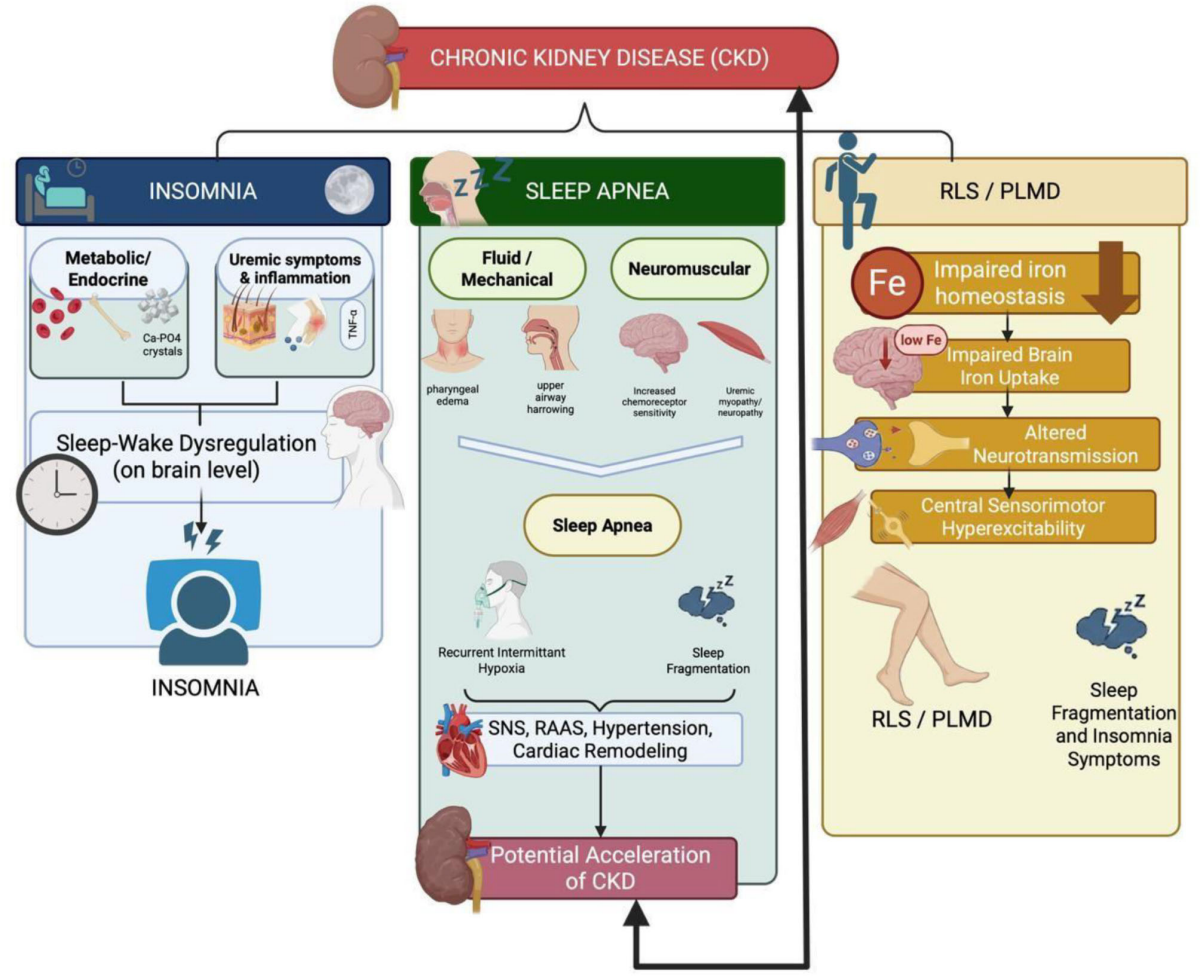
Schematic overview of the links between CKD and major sleep disorders. CKD-related metabolic, endocrine, and uremic disturbances promote brain sleep–wake dysregulation and insomnia (left). Fluid/mechanical and neuromuscular alterations drive sleep apnea, with recurrent hypoxia and sympathetic–cardiovascular activation that may accelerate CKD progression (center). Impaired iron homeostasis in CKD leads to altered neurotransmission and sensorimotor hyperexcitability, contributing to restless legs syndrome/periodic limb movement disorder (RLS/PLMD), sleep fragmentation, and insomnia symptoms (right). RAAS, renin–angiotensin–aldosterone system.

The pathogenesis of sleep disorders in CKD is multifactorial, reflecting a complex interplay of metabolic, neurohumoral, psychological, and treatment-related mechanisms that converge on sleep–wake dysregulation (Fig. 1).

Insomnia arises from metabolic disturbances such as anaemia and iron deficiency, which may impair oxygen delivery and neurotransmitter synthesis [40]; disordered mineral metabolism and secondary hyperparathyroidism, sometimes improved by parathyroidectomy [41, 42]; uremic symptoms, including pruritus, cramps, neuropathic pain, and bone pain [43, 44]; chronic systemic inflammation with elevated cytokines such as interleukin-1β, interleukin-6, interleukin-18, tumour necrosis factor-α, and C-reactive protein [45, 46]; adverse effects of commonly used medications [47]; and psychological comorbidities such as depression, anxiety, and illness-related distress. Dialysis scheduling, particularly very early morning and late-night sessions, may also misalign circadian rhythms and promote daytime inactivity and napping, reducing homeostatic sleep drive [48, 49]. Disturbances of circadian biology play a central role. In healthy individuals, melatonin exhibits a robust nocturnal rise. In contrast, in CKD and haemodialysis patients, melatonin rhythms are often blunted or phase-shifted, with reports of both reduced nocturnal levels and paradoxical accumulation of melatonin and its active metabolites [50, 51]. These anomalies likely reflect impaired renal clearance, uremic toxicity, and altered light–dark exposure, and contribute to circadian misalignment and sleep–wake instability.

**Figure 2:**
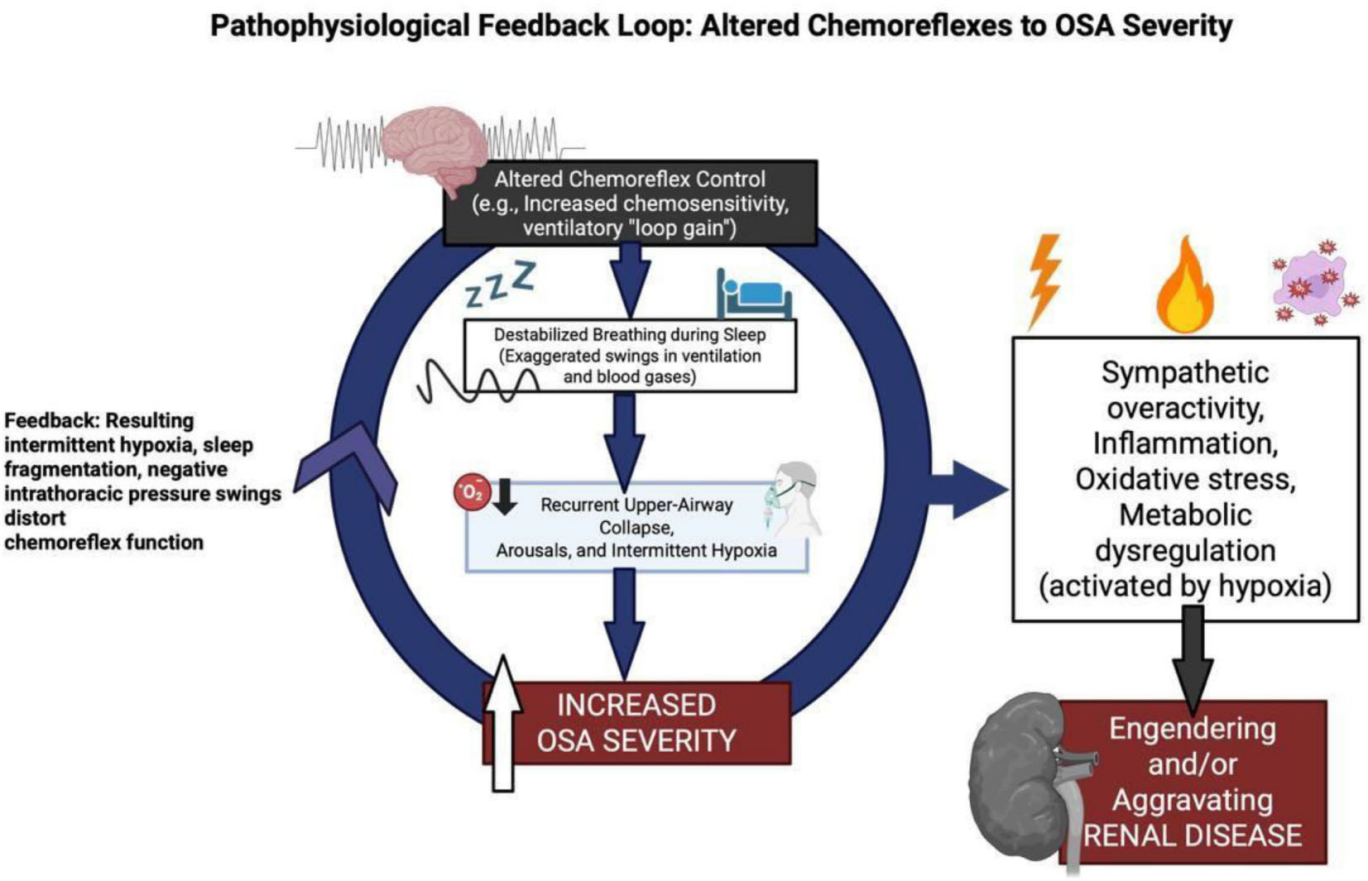
Pathophysiological feedback loop linking altered chemoreflexes, OSA, and renal disease. Heightened chemoreflex control destabilizes breathing during sleep, leading to recurrent upper-airway collapse, arousals, and intermittent hypoxia that increase OSA severity. The resulting intermittent hypoxia, sleep fragmentation, and intrathoracic pressure swings further distort chemoreflex function, while hypoxia-driven sympathetic activation, inflammation, oxidative stress, and metabolic dysregulation promote the onset and/or progression of renal disease.

The pathogenesis of sleep apnoea in CKD is similarly multifaceted. The pathophysiological details linking OSA to kidney disease are illustrated in Fig. 2. Chronic hypervolemia and extracellular fluid overload cause fluid accumulation in the legs during the day and rostral fluid shift at night, leading to peripharyngeal tissue oedema and increased upper airway collapsibility, which promote OSA [52, 53]. Pulmonary congestion and interstitial oedema can stimulate pulmonary receptors and contribute to CSA [54]. Enhanced chemoreceptor sensitivity to hypoxia and hypercapnia destabilizes ventilatory control and fosters central apnoeas and Cheyne–Stokes-like breathing. Uremic myopathy and neuropathy may impair respiratory muscle endurance and upper airway dilator function. Interventional data support the causal role of fluid overload: intensified ultrafiltration and nocturnal dialysis, which improve volume control, reduce the apnea hypopnea index, and ameliorate sleep-disordered breathing in kidney failure [52, 55]. Recurrent apnoeas produce intermittent hypoxia, intrathoracic pressure swings, and sleep fragmentation, which, in turn, activate the sympathetic nervous system and the renin–angiotensin–aldosterone system, increase blood pressure and intraglomerular pressure, and promote oxidative stress, inflammation, and endothelial dysfunction [56]. These changes foster albuminuria, progressive tubulointerstitial fibrosis, and cardiovascular damage, plausibly accelerating CKD progression [56].

The pathophysiology of RLS and PLMD in CKD involves disturbances in iron homeostasis, dopaminergic neurotransmission, and excitatory–inhibitory balance within central sensorimotor circuits. Brain iron deficiency is a key pathogenic feature. In CKD, reduced systemic iron stores, chronic inflammation, and functional iron deficiency further compromise brain iron uptake [57, 58]. Dopaminergic alterations, with a presynaptic hyperdopaminergic state leading to postsynaptic D2 receptor downregulation, combined with the circadian decline of dopamine at night, create a relative nocturnal dopaminergic deficit [59, 61]. Hyperglutamatergic and hypoadenosinergic states contribute to cortical hyperarousal and insomnia symptoms. Medications such as certain antidepressants, antipsychotics, and prokinetic agents may precipitate or worsen RLS by disturbing dopaminergic and serotonergic balance. Peripheral neuropathy, common in advanced CKD, may provide aberrant sensory input that fuels RLS dysaesthesias. The high prevalence of RLS and PLMD in dialysis cohorts, and the frequent amelioration or resolution of symptoms after successful kidney transplantation, strongly suggest that uremia, toxin accumulation, and disordered iron metabolism are major upstream drivers.

## CLINICAL IMPLICATIONS FOR NEPHROLOGY AND HYPERTENSION PRACTICE

Although definitive causal evidence is limited, the available data justify a proactive approach to sleep disorders in patients with or at risk of CKD. Several practical implications emerge for clinical practice.

### Screening and case finding

Clinicians should routinely enquire about sleep quality, difficulty in initiating or maintaining sleep, nonrestorative sleep, snoring, witnessed apnoeas, nocturnal choking or dyspnoea, nocturia, RLS symptoms, and daytime sleepiness in patients with CKD or at high risk of CKD. Particular attention should be given to individuals with resistant or nocturnal hypertension, nondipping blood pressure profiles, obesity, type 2 diabetes, or unexplained albuminuria, in whom the pretest probability of OSA is high. Obesity plays multiple roles in the relationship between sleep disorders, particularly OSA, and CKD. It acts as a classic confounder because it is strongly associated with both OSA and CKD, but may also lie on the causal pathway, mediating part of the impact of OSA on CKD progression via haemodynamic, metabolic, and inflammatory mechanisms. In addition, obesity may modify the effect of OSA on renal outcomes, with obese patients potentially experiencing a greater renal risk burden from comparable degrees of sleep-disordered breathing than nonobese patients.

### Referral for sleep evaluation

A high index of suspicion for moderate–severe OSA—based on symptoms, validated questionnaires (e.g. STOP–BANG [62] or Epworth sleepiness scale [63]) and, where available, nocturnal oximetry—should prompt referral for formal sleep assessment. Polysomnography or high–quality home sleep testing is necessary to diagnose OSA and CSA accurately and to distinguish these conditions from insomnia or RLS–related sleep disruption. Given the consistent association of OSA with higher CKD risk and faster eGFR decline, identifying and treating OSA may have implications for kidney and cardiovascular health.

### Optimizing modifiable factors

Several CKD–related factors that contribute to sleep disorders are amenable to intervention. Optimizing volume status (e.g. by individualizing dry weight and dialytic ultrafiltration), promoting weight reduction, correcting iron deficiency, treating anaemia, rationalizing medications that worsen sleep or provoke RLS, and minimizing circadian disruption from very early or late dialysis shifts are pragmatic strategies that may improve both sleep and cardiorenal risk profiles. Addressing pain, pruritus, mood disorders, and physical inactivity can further enhance sleep quality.

### Therapeutic considerations

Small interventional and observational studies suggest that effective OSA therapy (e.g. CPAP or, in selected patients, surgery) may slow eGFR decline and reduce albuminuria, particularly in high–risk populations, although robust randomized evidence is lacking. Similarly, optimizing RLS management with iron supplementation and dopaminergic or α2δ–ligand therapy may improve sleep quality and patient–reported outcomes, even if a direct renoprotective effect has not been demonstrated. Cognitive behavioural therapy for insomnia represents a promising nonpharmacological option, but data specific to CKD are sparse.

### Patient communication

When discussing sleep disorders with patients, clinicians should emphasize that treatment is clearly justified for symptom relief, improvement in quality of life, and cardiovascular risk reduction. Potential kidney benefits are biologically plausible and supported by observational data, but not yet definitively proven; this uncertainty should be communicated transparently while still motivating systematic identification and management of sleep problems.

In clinical practice, OSA should be actively considered in CKD patients with resistant or difficult–to–control hypertension, nondipping or reverse–dipping blood pressure patterns, obesity (especially central obesity), or typical OSA symptoms (snoring, witnessed apnoeas, daytime sleepiness). In these subgroups, systematic clinical screening (e.g. questionnaires) and a low threshold for referral to sleep studies appear justified.

## CONCLUSION AND PERSPECTIVES

Sleep disorders are highly prevalent across the CKD spectrum and in individuals at high risk of CKD, and they appear to contribute to both CKD development and progression, as well as to excess cardiovascular morbidity and mortality. Prospective observational data suggest that poor sleep quality, insomnia symptoms, abnormal sleep duration, and, most consistently, OSA are associated with a higher risk of incident CKD, faster eGFR decline, albuminuria, and ESKD. However, residual confounding by shared cardiometabolic risk factors, the frequent coexistence of multiple sleep disorders and incomplete phenotyping of OSA in many studies mean that causal inferences remain tentative.

The strength of evidence linking individual sleep disorders to renal outcomes is not uniform. Among the conditions considered, OSA has the most consistent epidemiological support for an association with incident CKD, accelerated eGFR decline, and albuminuria, and is mechanistically linked to intermittent hypoxia, sympathetic activation, renin–angiotensin–aldosterone system activation, oxidative stress, and endothelial dysfunction—all plausible drivers of progressive kidney damage. By contrast, evidence that insomnia, RLS, or PLMD independently cause incident CKD or faster CKD progression is limited and largely observational, with substantial potential for confounding by coexisting OSA, comorbidities, and CKD severity. Mendelian randomization studies to date do not support a strong causal effect of OSA on renal function, but indicate that impaired kidney function may predispose to OSA.

From a clinical perspective, the available evidence supports a more systematic integration of sleep assessment into nephrology and hypertension care. Routine screening for sleep complaints, systematic consideration of OSA in patients with obesity or resistant or nocturnal hypertension or nondipping blood pressure patterns, and timely referral for sleep evaluation are warranted. Optimizing volume status, reducing weight, correcting iron deficiency, reviewing medications that disturb sleep or provoke RLS, addressing mood disorders, and adapting dialysis schedules where possible are pragmatic measures that improve sleep and mitigate cardiovascular and renal risk. Small interventional studies and observational cohorts suggest that OSA therapy, including CPAP and surgery, may slow eGFR decline and reduce albuminuria in selected patients. However, definitive evidence from large-scale, methodologically robust randomized trials with kidney endpoints is still lacking.

The most urgent research priority is adequately powered, multicentre RCTs testing CPAP or alternative OSA therapies in patients with pre-existing CKD (e.g. stages 2–4) and in high-risk individuals (e.g. with diabetes, hypertension, or obesity) who have preserved eGFR but OSA. These trials should incorporate thorough sleep phenotyping, standardized renal endpoints (including eGFR slope, albuminuria, and incident ESKD), adjudicated cardiovascular outcomes, and rigorous adherence monitoring. Short- and medium-term mechanistic studies in smaller CKD cohorts, focusing on blood pressure control, nocturnal dipping patterns, sympathetic activation, oxidative stress, endothelial dysfunction, and markers of tubulointerstitial injury, are also needed to clarify causal pathways and identify subgroups most likely to benefit from treatment.

A layered research agenda that combines mechanistic investigations, pragmatic outcome trials, and implementation studies is essential to determine whether systematic identification and treatment of sleep disorders, particularly OSA, should become an integral component of renal and cardiovascular risk management across the CKD spectrum.

## Data Availability

No new data were generated for this review.
